# Supplementary material for: Type VIIb secretion system recruits the dedicated cell wall hydrolase EssH to enable effector secretion by Staphylococcus aureus
Source: mBio. 2026 Feb 26;17(4):e03196-25. doi: 10.1128/mbio.03196-25 (PMC13059805; doi:10.1128/mbio.03196-25)
Supplement: Supplemental material — Tables S1 and S2; Figures S1 to S5. [file mbio.03196-25-s0001.pdf]

Supporting Information

Type VIIb secretion system recruits a dedicated cell wall hydrolase EssH to enable effector secretion by *Staphylococcus aureus*

Richard Agyen[a], Isabelle Powell[a], Mahalia McNair[c], Dominique Missiakas[c], Maksym Bobrovskyy[a,b]#

[a]Department of Molecular Biosciences, University of South Florida, Tampa, Florida, USA
[b]Center for Antimicrobial Resistance, University of South Florida, Tampa, Florida, USA
[c]Howard Taylor Ricketts Laboratory, Department of Microbiology, University of Chicago, Chicago, Illinois, USA

2 supporting tables, 5 figures with legends, and references list

Table S1. Strains and plasmids used in this study.

Strain or plasmid	Description	Source or reference
**Strains**		
S. aureus		
USA300 LAC*	USA300 LAC cured of pUSA03 herein referred as wild type	(1, 2)
RN4220	Restriction-deficient cloning intermediate	(3)
essH	USA300 LAC* *essH::ermB*	(4)
Δ*essB*	USA300 LAC* Δ*essB*	(5)
Δ*esaA*	USA300 LAC* Δ*esaA*	(6)
Δ*esxA-0304*	USA300 LAC* Δ*esxA-0304*	(7)
Δ*essH*	USA300 LAC* Δ*essH*	This study
essH$^{\Delta ND}$	USA300 LAC* *essH*$^{\Delta ND}$, nucleotides 114-438 deleted	This study
essH$^{\Delta CHAP}$	USA300 LAC* *essH*$^{\Delta CHAP}$, nucleotides 454-894 deleted	This study
AH1919	USA300 LAC* Δ*aur* Δ*sspAB* Δ*scpA* *spl::ermB* (Δproteases)	(8)
BDG2485	USA300 LAC* *aur::Tn::ermB*	(9)
BDG2487	USA300 LAC* *sspA*::Tn::*ermB*	(9)
BDG2488	USA300 LAC* *sspB*::Tn::*ermB*	(9)
BDG2486	USA300 LAC* *scpA*::Tn::*ermB*	(9)
DM2288	USA300 LAC* *spl::ermB*	(9)
E. coli		
DH5α	F⁻ φ80*lac*ZΔM15 Δ(*lac*ZYA-*arg*F)U169 *rec*A1 *end*A1 *hsd*R17(r_K^-, m_K^+) *pho*A *sup*E44 λ⁻ *thi*-1 *gyr*A96 *rel*A1	Our collection
BL21	B F⁻ *ompT gal dcm lon* $hsdS_B(r_B^- m_B^-)$ [*malB*⁺]$_{K-12}$(λ^S)	(10)
**Plasmids**		
pSEW016	pWWW412 with a modified polylinker herein referred as vector	(4, 11)
pET15b	*E. coli* vector for production of recombinant proteins	Addgene
p*essH*	*essH* open reading frame cloned into pSEW016	(4)
p*essH*$^{C199A/H254A}$	*essH*$^{C199A/H254A}$ cloned into pSEW016	(4)
p*essH*$^{\Delta ND}$	*essH* nucleotides 1-73 and 454-894 are fused and cloned into pSEW016	This study

p$_{\text{STREP}}$*essH*	*S. aureus* USA300 LAC *essH* with Strep-tag II sequence inserted between nucleotides 72 and 73, and cloned into pSEW016	(4)
p*essC*	*S. aureus* USA300 LAC *essC* cloned into pSEW016	(7)
p*essC*$_{TS}$	*S. aureus* USA300 LAC *essC* with C-terminal Twin Strep-tag (TS) cloned into pSEW016	(7)
pET15b-$_{\text{STREP}}$*essH*	*essH* nucleotides 73-894 with N-terminal Strep-tag II cloned into pET15b	(4)
pET15b-$_{\text{STREP}}$*essH*$^{\Delta \text{CHAP}}$	*essH* nucleotides 73-453 with N-terminal Strep-tag II cloned into pET15b	This study
pET15b-$_{\text{STREP}}$*essH*$^{\Delta ND}$	*essH* nucleotides 454-894 with N-terminal Strep-tag II cloned into pET15b	This study

Table S2. Primers used in this study.

Primer	Sequence (5'→3')
MKP2R	GGGGACCACTTTGTACAAGAAAGCTGGGTCGACTAAGTTTTTGTTAAAGTCGTTAAGTACATTACTTACAAC
MMP1F	GGGGACAAGTTTGTACAAAAAAGCAGGCTCGTTATATGTCGTTGGTTGATCCTCGTTAAC
MMP1R2	CTGGTCGCTTTTTATTGATGTCATGATTAATTTAGTCTAAATCGATGCCTGTAATAAGCCCTTTGTTATCTTTTGAAAAAGCG
MMP2F	TAAATTAATCATGACATCAATAAAAAGCGACCAGTTCG
MMP2R	GGGGACCACTTTGTACAAGAAAGCTGGGTGCTTTTGGAGGAATATTTTATATAAGTCTAATAGTTCCTGTAACAC
MBP37F	CCCCGAGCTCATGAAGAAAACAATTTTACTGACGATGACAACTC
MBP37R	CCCCGGATCCTTAATGGATGTAATTATATGATGAAACTTCTGAAGCAGAG
MBP45F	CCCCCCATGGGCTGGTCACATCCTCAATTCGAAAAATATACGAATGATAGCAAAACATTAGAAGAAGCAAAG
MBP61R	TTTTTCGAATTGAGGATGTGACCACGTATATGCTTGAGCCGAGTTAGGCG
MBP70F	CTCGGCTCAAGCATATACGGACGAATTGTATGACGAATTACAAATCGCC
MBP149F	CCCCCCATGGGCTGGTCACATCCTCAATTCGAAAAAGAATTGTATGACGAATTACAAATCGCCGAATTTAATG

MBP186F	GGGGACAAGTTTGTACAAAAAAGCAGGCTGTGAAAATCCTAATCCTGCAGTTAATGCCATAG
MBP186R	TCATATAATTACATCCATTAAATTAATCATGACATCAATAAAAAGCG
MBP187F	GATGTCATGATTAATTTAATGGATGTAATTATATGACTTCATAATAACCATTTAATCCTTTATGTATTTAATTTAATTTTAGTATAC
MBP187R	GGGGACCACTTTGTACAAGAAAGCTGGGTGTACATTACTTACAACTTTTTCTGTGTTTTTAGCTACTTC
MBP199F	GGGGACAAGTTTGTACAAAAAAGCAGGCTGGCGCTCGCTTTGTTGATCATGATTTAAG
MBP199R	GGCATCGATTTAGACGAATTGTATGACGAATTAC
MBP201F	GTAATTCGTCATACAATTCGTCTAAATCGATGCCTGCTTTCTTTGCTTCTTCTAATGTTTTGCTATC
MBP212R	GGGGGGATCCAATGTCTAAATCGATGCCTGTAATAAGCCCTTTG

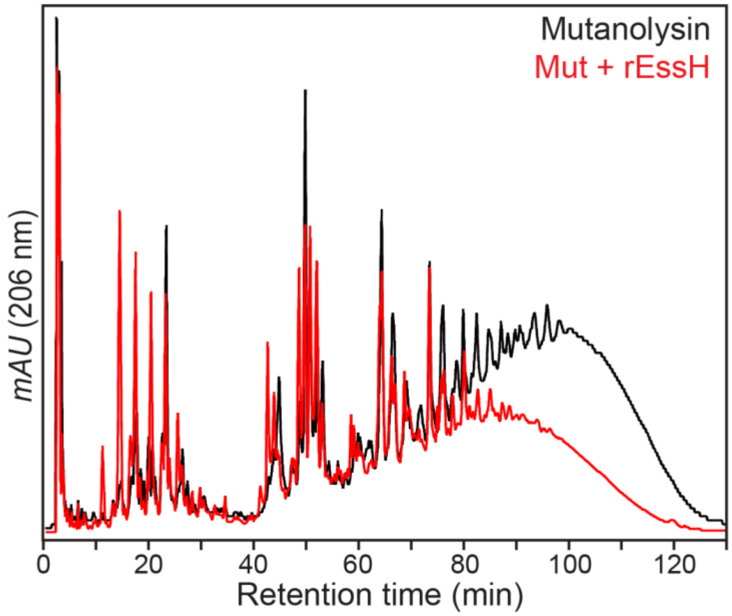

Figure S1. Purified rEssH has PG hydrolytic activity. BL21 pET15b-STREP*essH* was cultured and production of rEssH was induced with 1mM IPTG. Cells were lysed and soluble fraction applied to Strep-tactin Sepharose resin to purify rEssH by affinity chromatography. Murein sacculi were purified from *S. aureus* USA300 LAC* and treated with mutanolysin before digestion with rEssH or buffer alone as control. Resulting samples were resolved by C^{18} reversed-phase HPLC and Absorbance at 206 nm wavelength measured.

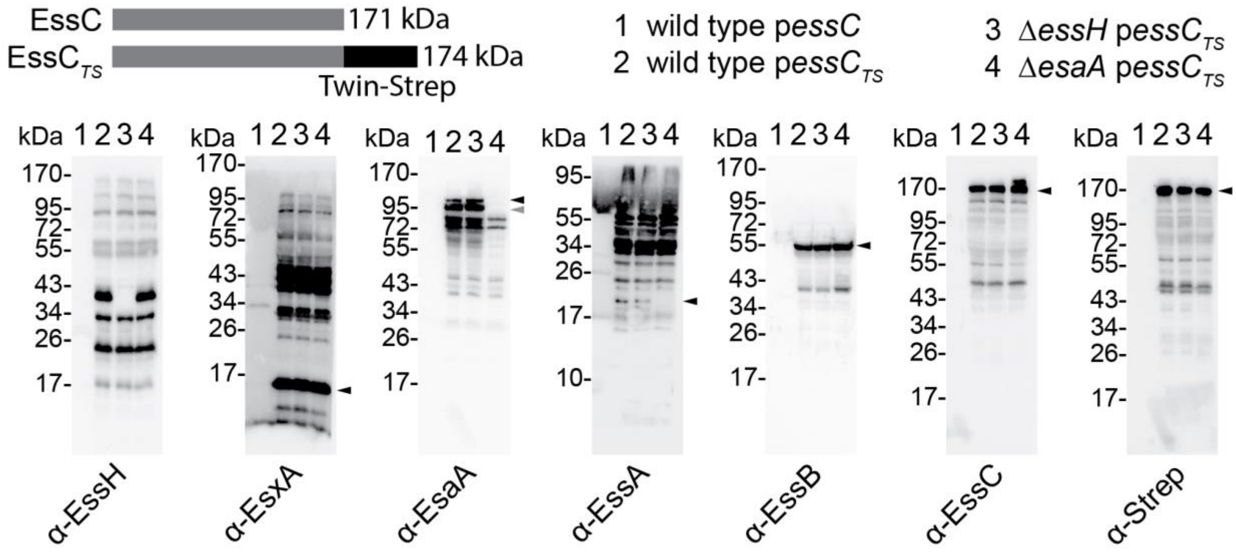

Figure S2. **EssH co-purification with the T7SSb complex.** T7SSb complex was purified by affinity chromatography from detergent-soluble fraction of *S. aureus* wild type, Δ*essH* and Δ*esaA* expressing *essC*$_{TS}$ or wild type expressing *essC* as a control. Bacteria were cultured under T7 permissive conditions and detergent extracts from the membranes were prepared and purified using affinity chromatography over Strep-Tactin Sepharose resin. Eluted proteins were subjected to Western blot analysis using indicated polyclonal antibodies for detection of T7SSb proteins. Black arrows indicate immunoreactive species corresponding to the expected size of the proteins of interest. Grey arrow indicates an alternative immunoreactive EsaA species. Numbers (in kDa) indicate the migratory positions of molecular weight markers. Experiment was performed in triplicate and representative western blots are shown.

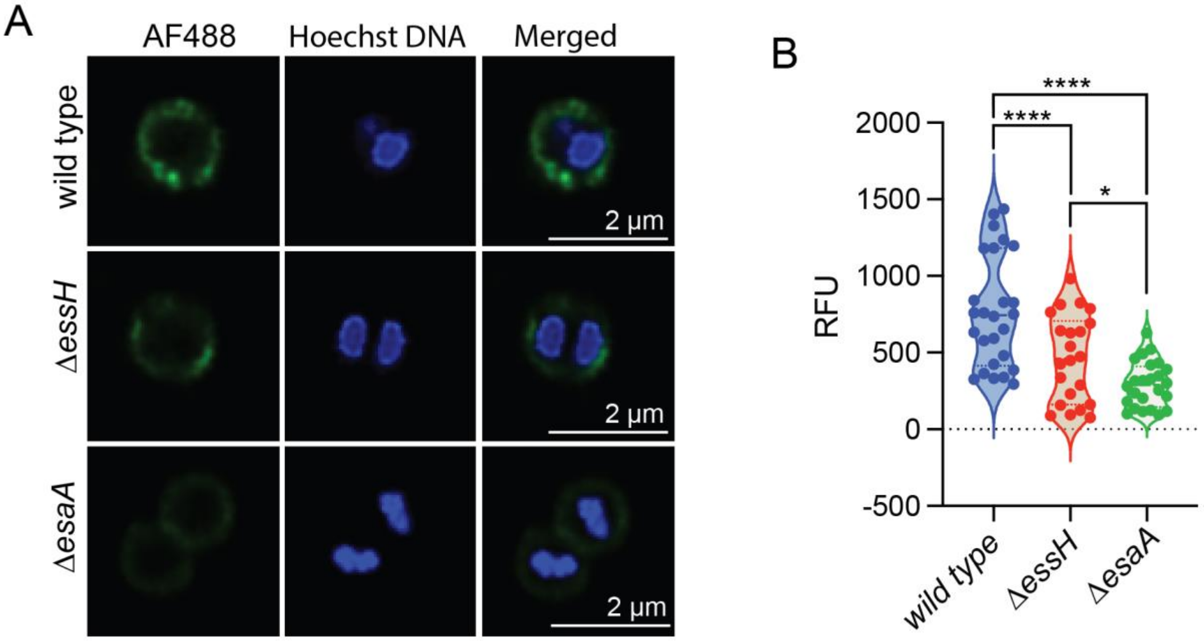

Figure S3. EssH is necessary for surface display of EsaA. A) Wild type, Δ*essH* and Δ*esaA* strains were washed, fixed and placed on the glass slide. To immunostain, cells were first blocked with BSA and monoclonal human IgG, then incubated with polyclonal rabbit serum specific for EsaA followed by washing and incubation with anti-rabbit monoclonal antibody conjugated to Alexa Fluor 488 (AF488). DNA was stained with Hoechst DNA dye and staphylococci were imaged using Nikon Eclipse Ti2 scanning confocal microscope and representative images shown. **B)** Fluorescence (RFUs) of >20 cells randomly selected from micrographs collected in three independent experiments in (A) was measured using Fiji software and plotted. Statistical significance was determined by ordinary one-way ANOVA with Tukey's multiple comparisons test; p-value <0.0332 (*), 0.0002(***), <0.0001(****).

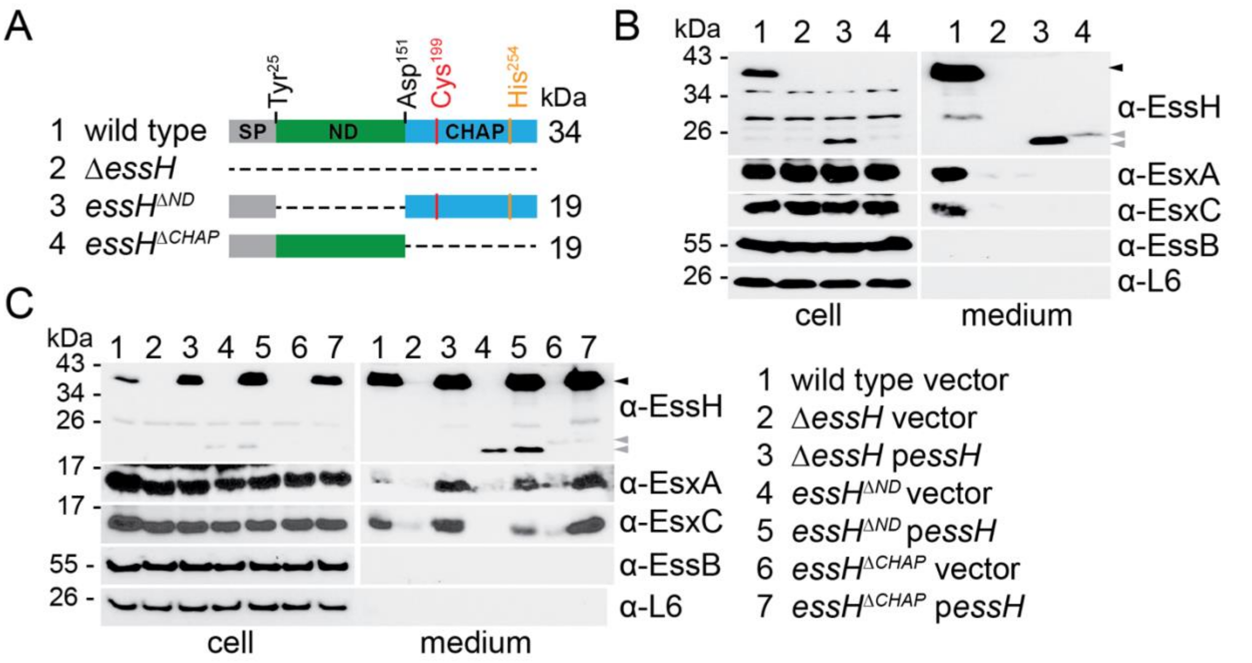

Figure S4. EssH ND and CHAP are required to support T7b secretion of EsxA and EsxC. A) Illustration of *essH* alleles encoded by wild type, Δ*essH*, *essH*$^{\Delta ND}$, *essH*$^{\Delta CHAP}$ strains. **B)** Strains from (A) were cultured under T7 permissive conditions to OD_{600} 3.0. Cultures were fractionated and analyzed by western blot using polyclonal rabbit antibodies specific for EssH (α-EssH), EsxA (α-EsxA), EsxC (α-EsxC), EssB (α-EssB) and L6 (α-L6). **C)** Wild type and Δ*essH*, *essH*$^{\Delta ND}$, *essH*$^{\Delta CHAP}$ mutants harboring either vector or p*essH* were analyzed as in (B).

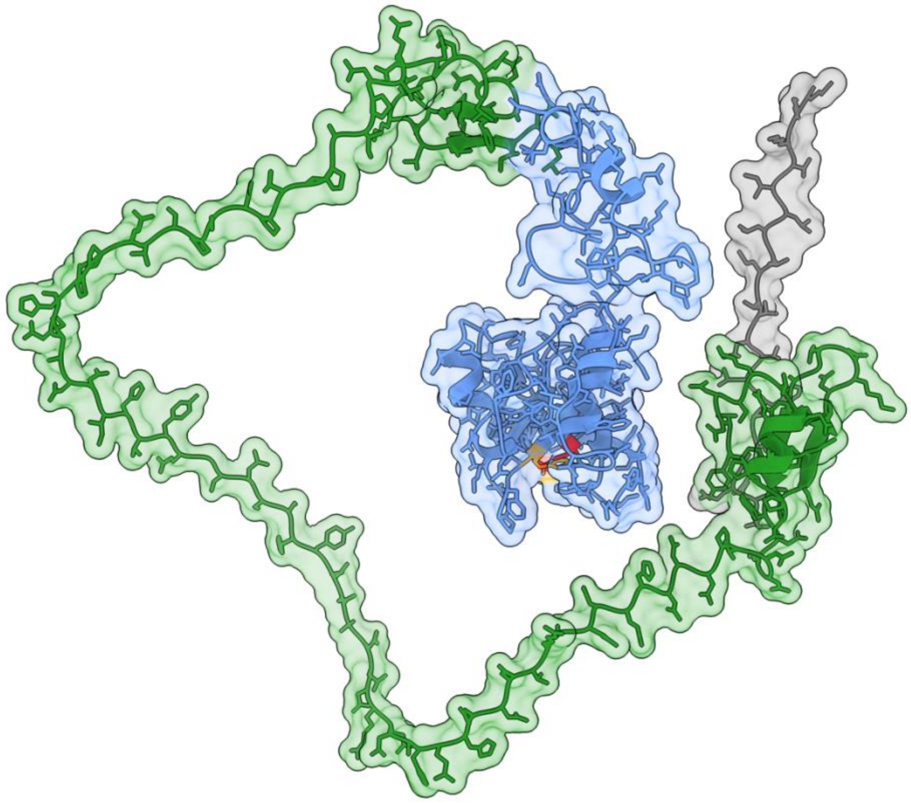

Figure S5. Structure of EssH predicted by AlphaFold 3. Signal peptide is highlighted in gray, N-terminal domain (ND) in green and CHAP domain in blue. Cys^{199} (red) and His^{254} (yellow) comprise the active site of the CHAP domain.

References

1. Diep BA, Gill SR, Chang RF, Phan TH, Chen JH, Davidson MG, Lin F, Lin J, Carleton HA, Mongodin EF, Sensabaugh GF, Perdreau-Remington F. 2006. Complete genome sequence of USA300, an epidemic clone of community-acquired meticillin-resistant Staphylococcus aureus. Lancet 367:731-9.
2. Burts ML, DeDent AC, Missiakas DM. 2008. EsaC substrate for the ESAT-6 secretion pathway and its role in persistent infections of Staphylococcus aureus. Mol Microbiol 69:736-46.
3. Kreiswirth BN, Lofdahl S, Betley MJ, O'Reilly M, Schlievert PM, Bergdoll MS, Novick RP. 1983. The toxic shock syndrome exotoxin structural gene is not detectably transmitted by a prophage. Nature 305:709-12.
4. Bobrovskyy M, Willing SE, Schneewind O, Missiakas D. 2018. EssH Peptidoglycan Hydrolase Enables Staphylococcus aureus Type VII Secretion across the Bacterial Cell Wall Envelope. J Bacteriol 200.
5. Anderson M, Chen YH, Butler EK, Missiakas DM. 2011. EsaD, a secretion factor for the Ess pathway in Staphylococcus aureus. J Bacteriol 193:1583-9.
6. Aly KA, Anderson M, Ohr RJ, Missiakas D. 2017. Isolation of a membrane protein complex for type VII secretion in *Staphylococcus aureus*. J Bacteriol 199:e00482-17.
7. Bobrovskyy M, Oh SY, Missiakas D. 2022. Contribution of the EssC ATPase to the assembly of the type 7b secretion system in Staphylococcus aureus. J Biol Chem 298:102318.

8. Wormann ME, Reichmann NT, Malone CL, Horswill AR, Grundling A. 2011. Proteolytic cleavage inactivates the Staphylococcus aureus lipoteichoic acid synthase. J Bacteriol 193:5279-91.

9. Gimza BD, Jackson JK, Frey AM, Budny BG, Chaput D, Rizzo DN, Shaw LN. 2021. Unraveling the Impact of Secreted Proteases on Hypervirulence in Staphylococcus aureus. mBio 12.

10. Studier FW, Moffatt BA. 1986. Use of bacteriophage T7 RNA polymerase to direct selective high-level expression of cloned genes. J Mol Biol 189:113-30.

11. Bubeck Wardenburg J, Williams WA, Missiakas D. 2006. Host defenses against *Staphylococcus aureus* infection require recognition of bacterial lipoproteins. Proc Nat Acad Sci USA 103:13831-13836.
